# The Effect of Herbal Feed Additives in the Diet of Dairy Goats on Intestinal Lactic Acid Bacteria (LAB) Count

**DOI:** 10.3390/ani12030255

**Published:** 2022-01-21

**Authors:** Joanna Foksowicz-Flaczyk, Jacek Antoni Wójtowski, Romualda Danków, Przemysław Mikołajczak, Jan Pikul, Agnieszka Gryszczyńska, Zdzisław Łowicki, Karolina Zajączek, Daniel Stanisławski

**Affiliations:** 1 Department of Innovative Biomaterials and Nanotechnologies, Institute of Natural Fibres and Medicinal Plants, Wojska Polskiego 71b, 60-630 Poznań, Poland; joanna.flaczyk@iwnirz.pl; 2 Department of Animal Breeding and Product Quality Assessment, Faculty of Veterinary Medicine and Animal Science, Poznań University of Life Science, Złotniki, ul. Słoneczna 1, 62-002 Suchy Las, Poland; 3 Department of Dairy and Process Engineering, Faculty of Food Science and Nutrition, Poznań University of Life Sciences, ul. Wojska Polskiego 31/33, 60-624 Poznań, Poland; romualda.dankow@up.poznan.pl (R.D.); jan.pikul@up.poznan.pl (J.P.); 4 Department of Pharmacology and Phytochemistry, Institute of Natural Fibres and Medicinal Plants, Kolejowa 2, 62-064 Plewiska, Poland; przemmik@ump.edu.pl (P.M.); agnieszka.gryszczynska@iwnirz.pl (A.G.); zdzislaw.lowicki@iwnirz.pl (Z.Ł.); karolina.zajaczek@iwnirz.pl (K.Z.); 5 Department of Pharmacology, Poznań University of Medical Sciences, ul. Rokietnicka 5a, 60-806 Poznań, Poland; 6 Computer Laboratory, Poznań University of Life Sciences, ul. Wołyńska 33, 60-637 Poznań, Poland; daniel.stanislawski@up.poznan.pl

**Keywords:** herbal feed additives, intestinal lactic acid bacteria (LAB), dairy goats

## Abstract

**Simple Summary:**

The prohibition on the use of antibiotics in animal nutrition has resulted in the more frequent use of phytobiotics, which are natural medical preparations made from herbs. When used in the nutrition of ruminants, phytobiotic preparations affect the motility of the gastrointestinal tract and the secretion of digestive juices, and also stimulate the development of the intestinal microbiota. Their effect on the development of lactic acid bacteria (LAB), with subsequent effects on the degree of microbial homeostasis in the gastrointestinal tract, is particularly important. The aim of the present study was to evaluate the effects of herbal supplements on lactic acid bacteria (LAB) count in the faeces of lactating dairy goats. It was assumed that the specific chemical composition of herbal supplements would positively affect the digestive processes of does, and thus the growth of lactic acid bacteria (LAB) colonies. The research was conducted on dairy goats assigned to five nutrition groups of twelve animals each. The animals in the experimental groups received a supplement made of (seven or nine) herbs at a rate of 20 g or 40 g per animal per day. A statistically significant effect of lactation stage on the intestinal *Lactobacillus* bacteria count was found. The highest concentration of LAB was found in the group receiving a feed supplement consisting of nine herbs at 20 g per animal per day. A probiotic strain of *Lactobacillus fermentum* absent from the control goats was identified in the faecal samples of goats that receiving the herbal supplement.

**Abstract:**

Sixty dairy goats of the Polish white improved breed were randomly assigned to five feeding groups of twelve animals each. The animals received a supplement containing seven herbs at 20 or 40 g/animal/day (experimental groups 1 and 2) and a supplement containing nine herbs at 20 or 40 g/animal/day (experimental groups 3 and 4)m, along with pelleted concentrate feed. Group 5 (the control group) received pelleted feed without any herbal supplements. A significant effect of herbal feed additive on lactic acid bacteria (LAB) count was observed (*p* < 0.001). The highest number density of LAB was found in the goats receiving the feed additive with nine herbs at 20 g/animal per day (*p* < 0.05). There was a statistically significant effect of lactation stage on intestinal LAB count (*p* < 0.001). Regardless of the feeding group, the highest number density of LAB was found in animals at the peak of lactation. The LAB count was also affected by the interaction of diet group × lactation stage (*p* < 0.0001). A probiotic strain of *Lactobacillus fermentum* was identified in the faecal samples of goats receiving the herbal additive, but not in the controls. Genetic identification of the microorganisms isolated from the faeces of the experimental goats did not reveal the presence of harmful mould spores, although spores of the fungus Aspergillus fumigatus were detected in the controls.

## 1. Introduction

The prohibition on the use of antibiotics in animal nutrition has resulted in the increased use of natural substances derived from medicinal plants [1]. Herbs containing bioactive ingredients—phytobiotics—have a particularly broad spectrum of action [2,3].

Phytobiotic mixtures are produced from wild plants or extracted from field crops [4]. Herbal raw materials are those parts of plants in which the accumulation of active ingredients is relatively high, and may include leaves, rhizomes, roots, flowers, bark, fruit, or seeds. The stimulating or prophylactic and therapeutic properties of plants are determined by their bioactive ingredient content, which is maximized by harvesting at the optimal vegetative phase, the appropriate conditions and place of harvesting, proper drying, and storage [5]. Even under proper storage conditions, the properties intensity of the active ingredients of herbs diminishes over time [6]. Production waste from the herbal industry may also be used as a feed additive, provided that it still has an appropriate active ingredient content. One example is the endosperm of milk thistle, which is a waste product in the production of silymarin [7]. The most important groups of bioactive ingredients that are found in herbal raw materials are tannins, saponins, essential oils, flavonoids, glycosides, and alkaloids [8].

The phytobiotic preparations used in feeding domestic animals, especially ruminants, can enhance taste sensations and stimulate appetite. As regulators of digestive functions, they also affect gastrointestinal (GI) motility and the secretion of digestive juices, reduce the occurrence of diarrhoea, and regulate the pH of the GI tract [9]. Some of them may also have a protective effect (such as fenugreek and flax), regulate metabolism (e.g., knotgrass, nettle), or affect the quality of animal products (e.g., garlic and calendula flower) [10].

Animals’ taste preferences should also be taken into consideration when formulating herbal mixes. Herbs usually contain high levels of essential oils and there may be problems with their uptake by some ruminants, such as sheep [11].

The herbal feed additives provided to ruminants stimulate digestive processes by supporting rumen microorganisms [9,12]. In particular, their effect on the growth of probiotic LAB is important, because it affects the degree of gastrointestinal (GI) microbial homeostasis. The gut flora balance constitutes an effective barrier against pathogen colonisation, influences the production of metabolic substrates (e.g., vitamins and short-chain fatty acids), and positively stimulates the immune system [13].

There are more than 180 species of *Lactobacillus,* and these include the homofermentative and mesophilic *Lactococcus lactis*, the best-known species of LAB. Strains with proven probiotic properties are considered most valuable, and include *Lactobacillus rhamnosus*, *Lactobacillus casei*, *Lactobacillus bulgaricus*, *Lactobacillus salivarius*, *Lactobacillus plantarum*, *Lactobacillus acidophilus* and *Lactobacillus helveticus* [14].

This study aimed to evaluate the effects of herbal supplements incorporated into the diets of lactating dairy goats on faecal LAB count. We assumed that the specific chemical composition of the herbal supplements would have a positive effect on the digestive processes of the animals, thus increasing the colonies of LAB that fortify microbial homeostasis in the GI tract.

## 2. Materials and Methods

### 2.1. Ethical Approval

All the research was performed in accordance with the Polish Act on the protection of animals used for scientific or teaching purposes, which complies with EU legislation on the protection of animals used for scientific purposes. All procedures were approved by the Local Bioethics Committee for Animal Testing (Poznań, Poland; decision no. 57/2020).

### 2.2. Location and Animal Material

The experiment was conducted on sixty Polish white improved goats kept on a specialised farm located in northwestern Poland (Bukowiec, 52°51′41″ N; 16°52′12″ E) in the Wielkopolska region. Clinically healthy goats were selected for the experiment. The somatic cell count (SCC) measured immediately prior to the experiment (during the third week of lactation) was at an acceptable level, and did not exceed 800 × 10^3^/mL. The animals were aged 20–30 months, were in their second lactation, and had a body weight of 56–60 kg. The experiment started when goats were approximately 28.1 ± 2.7 days in milk (DIM).

The animals were randomly assigned to five feeding groups of twelve goats each:

Group 1 (receiving 20 g of herbal supplement—mix of seven herbs).
Group 2 (receiving 40 g of herbal supplement—mix of seven herbs).
Group 3 (receiving 20 g of herbal supplement—mix of nine herbs).
Group 2 (receiving 40 g of herbal supplement—mix of nine herbs).
Group 5 (control group, no herbal supplements).

The amount of herbal supplement provided (20 g or 40 g/goat/day) was based on the experiment of Jarzynowska and Peter [15] on dairy sheep.

Goats were tagged using electronic transponders and coloured collars, varied by group, with plastic numbers.

### 2.3. Herbal Supplements

The herbal supplements provided to the experimental goats were composed of seven herbs (herbal supplement I) or nine herbs (herbal supplement II). The choice of herbs for the supplements was established on the basis of our previous experiments (unpublished) and herbs in supplement for dairy sheep in the Jarzynowska and Peter’s experiment [15].

Herbal mix 1 included common nettle *Urtica dioica* L. (herb); common agrimony *Agrimonia eupatoria* (herb—dried flowering shoot tips); caraway *Carum carvi* (fruit); coriander *Coriandrum sativum* (fruit); fenugreek *Trigonella foenum graecum* L. (seeds); plantain *Plantago lanceolata* L. (herb); and purple willow *Salix purpurea* (bark).

Herbal mix 2 contained different proportions of herbs to that used in herbal mix 1. The herbs included were common nettle *Urtica dioica* L. (herb); common agrimony *Agrimonia eupatoria* (herb—dried flowering shoot tips); coriander *Coriandrum sativum* (fruit); fenugreek *Trigonella foenum graecum* L. (seeds), as well as fennel *Foeniculum vulgare* (fruit); peppermint *Mentha piperita* (leaves); chamomile *Matricaria chamomilla* L. (flower clusters); milk thistle *Silybum marianum* (endosperm); and thyme *Thymus vulgaris* (leaves).

A detailed contribution of particular herbal components were included in these supplements is presented in our patent applications (Polish Patent Office submissions P.4334426 and P.433779)

### 2.4. Animal Nutrition

The diets were formulated to meet the animals’ nutrient requirements: 2.12 UFM (unit for milk production) and 185 g PDI (protein truly digestible in the small intestine) to obtain an assumed milk yield of 3.0 kg and 3.8% of fat [16].

The ingredients (% DM) of diet offered to dairy goats were:

15.6% maize silage; 21.6% grass hay silage; 7.8% brewers’ grain silage; 26.4% concentrate mixture; 10.3% meadow hay; 10.6% experimental concentrate (with herbal mix); 4.4% dried sugar beet pulp; and 3.3% barley straw.

The chemical composition of the diet was 451 g kg^−1^ of DM organic matter, 163 g kg^−1^ of DM crude protein, 267 g kg^−1^ of DM acid detergent fibre (ADF), and 401 g kg^−1^ of DM neutral detergent fibre (NDF).

All the ingredients, other than the experimental concentrate with the herbal mix, were part of the total mixed ration (TMR) feed and were offered to the animals once a day. Goats had free access to water and a mineral salt lick.

The herbal supplement was provided to the animals in the prepared pelleted concentrate feed (cereal grains, rapeseed meal, sunflower meal), containing the concentration of the mixes of seven herbs (Group 1 and Group 2) and nine herbs (Group 3 and Group 4). Group 5 was a control group and thus did not receive the herbal supplement.

Group 1 (G1): basal diet plus 20 g DM herbal mix 1 in 300 g of concentrate (herbal mix 1, 6.6 g of 100 g^−1^ concentrate dry matter);
Group 2 (G2): basal diet plus 40 g DM herbal mix 1 in 300 g of concentrate (herbal mix 1, 13.2 g of 100 g^−1^ concentrate dry matter);
Group 3 (G3): basal diet plus 20 g DM herbal mix 2 in 300 g of concentrate (herbal mix 1, 6.6 g of 100 g^−1^ concentrate dry matter);
Group 4 (G4): basal diet plus 40 g DM herbal mix 2 in 300 g of concentrate (herbal mix 1, 13.2 g of 100 g^−1^ concentrate dry matter);
Group 5 (CTRL): basal diet plus 300 g concentrate (no herbs; control group).

The composition of experimental concentrates is shown in Table 1.

### 2.5. Microbiological Tests of Faeces

The faeces underwent microbiological testing to determine the amount of LAB, in order to assess the effects of the herbs on the microbiota of the digestive tract of the dairy goats. The faeces for testing were collected from the animals of the five groups at four times: before the start of the experiment (T0) and at the end of the first (T1), second (T2), and third (T3) trimesters of lactation. Faecal samples were collected directly from the previously disinfected milking stall floor during morning milking of each animal. Faeces were collected in individually labelled, sterile, 50 mL plastic containers. All samples were placed in an ice thermostat and were transported to the laboratory within two hours. Each collected sample (10 g) were individually dissolved in 10 g of sterile saline and shaken using a vortex mixer for 1 h; they were then diluted using the decimal dilution method and plated on Petri plates. The Petri plates were flooded with MRS agar broth nutrient medium containing 20.0 g/L agar, 20.0 g/L glucose, 10.0 g/L peptone K, 8.0 g/L Lab-Lemco powder, 4.0 g/L yeast extract, 1 mL sorbitan monooleate, 2.0 g/L dipotassium hydrogen phosphate, 5.0 g/L sodium acetate, 2.0 g/L triammonium citrate, 0.2 g/L magnesium sulphate, and 0.05 g/L manganese sulphate. The Petri plates were placed in an incubator and incubated under anaerobic conditions at 35–37 °C for 48–72 h. After the incubation, the number of single bacterial colonies grown on the plates was determined.

#### Identification of LAB Strains

After incubation, a similar number of samples from all experimental dates were collected from both the aerobically and anaerobically cultured samples. The cultured LAB strains were then identified.

Genetic material (DNA) was isolated from the most frequently and morphologically repetitive LAB colonies. Identification of LAB strains involved the following stages:

(1) Isolation of DNA from colonies grown on the plates.

Twelve isolations of genetic material (DNA) were performed on colonies of microorganisms provided on plates. DNA was isolated using CHELEX (Bio-Rad, Hercules, CA, USA) with the addition of enzymes to digest the cell wall.

(2) Amplification of the 16SrRNA gene fragment using polymerase chain reaction (PCR) with specific primers and sequencing of PCR arrays.

To confirm the presence of bacteria in the sample, amplification by PCR of 16S rDNA query fragments was performed using specific primers:

27F: 5-AGAGTTTGATCMTGGCTCAG-3;
1492R: 5-GGTTACCTTGTTACGACTT-3;
on the DNA template isolated from the colony.

The amplification reaction was performed in the ABI 9700 thermocycler (Life Technologies, Waltham, MA, USA) using thermostable OptiTaq polymerase (Eurx, Gdansk, Poland).

PCR conditions:

(1) 95 °C for 3 min.
(2) 95 °C for 15 s.
(3) 55 °C for 15 s.
(4) 72 °C for 90 s.
(5) Steps 2–4 were repeated 30 times.
(6) 72 °C for 2 min.
(7) 10 °C until cooled.

All the samples proved positive for amplification. PCR products were then purified, and sequencing was performed using a BigDye Terminator Mix v3.1 kit (Applied Biosystems, Forest City, CA, USA), an ABI3730xl genetic analyser, and specific primers.

The reads (from the bacterial 16S-rDNA-specific primers 27F and 1492R) were assembled into contigs, yielding a consensus sequence.

(3) Amplification by PCR of the internal transcribed spacer (ITS) fragment using specific primers and sequencing of PCR arrays.

To determine if there were any fungi present in the sample, amplification by PCR of ITS fragments was performed using specific primers:

ITS1: 5-TCCGTAGGTGAACCTGCGG-3;
ITS4: 5-TCCTCCGCTTATTGATATGC-3;
on the DNA template isolated from the colony.

All samples proved positive for amplification. The PCR products were then purified, and sequencing was performed using the BigDye Terminator Mix v3.1 kit, ABI3730xl genetic analyser, and specific primers. The reads from the ITS primers (ITS1-F and ITS4-R) were assembled into appropriate contigs, yielding a consensus sequence.

(4) Alignment of the obtained sequences and the NCBI database.

The consensus sequences were compared with the NCBI database (GeneBank, https://www.ncbi.nlm.nih.gov/gene/?term=, accessed on 25 October 2017) using the BLAST software (NCBI, Bthesda, MD, USA).

### 2.6. Statistical Analysis

**Figure 1:**
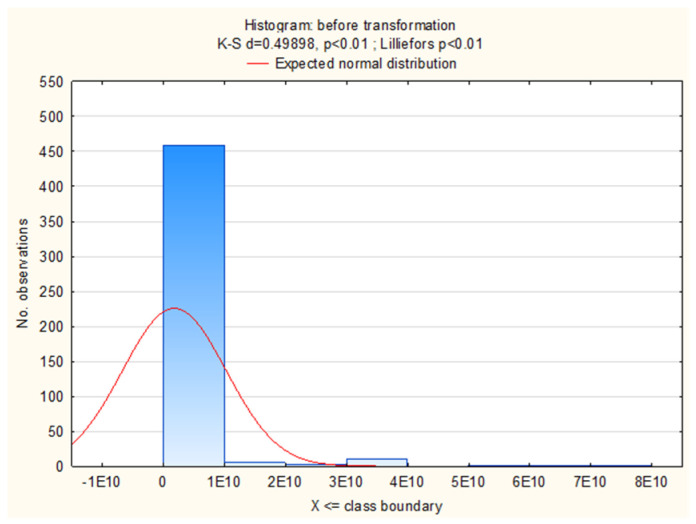

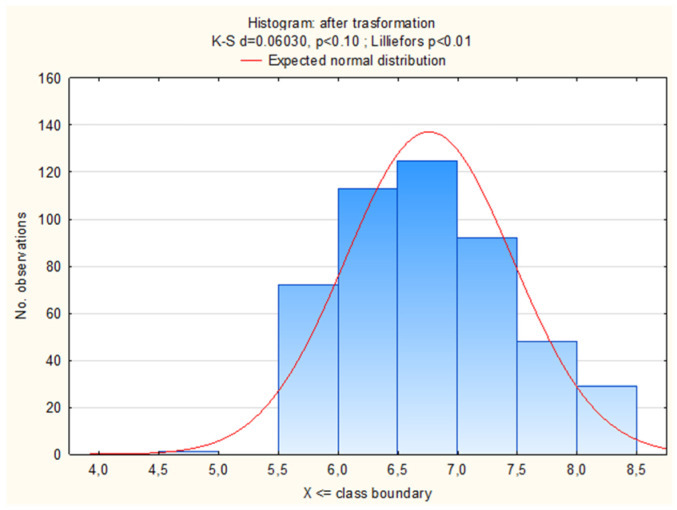
The distribution of LAB count before and after the Box–Cox transformation.

Data were analyzed using SAS version 9.4 (2014, SAS Institute, Cary, NC, USA). Before analysis was conducted, all the data were evaluated for normality using PROC UNIVARIATE SAS (SAS Institute, Cary, NC, USA). As no normal distribution was found in the collected samples (Figure 1), the count of LAB determined in the faeces underwent the Box–Cox transformation with an estimated λ = −0.114851.

Data were analysed using a PROC MIXED model (version 9.4, SAS Institute, Cary, NC, USA). The lowest Akaike Information Criterion (AIC) was used to determine the appropriate within-subject covariance structure, and the compound symmetry (CS) was selected accordingly. Data were analysed as repeated measures (goat effect) using the following model: Yjk = μ + gi + tj + tgij + eijk, where: Yijk Y_jk_ = μ + g_i_ + t_j_ + tg_ij_ + eijk, where the Y_ijk_ are the observation means, μ is the overall mean, the g_i_ are the fixed effects of the groups (l = 1, 2, 3, 4, 5), the t_j_ are the fixed effects of the time of measurement (k = 1, 2, 3, 4), the tg_ij_ are the interaction of group × time, and the e_ijk_ are the residual errors.

When differences were detected in terms of treatment or interactions of treatment with time, separation of means was conducted using a Tukey’s adjustment for the probability. The statistical significance was considered to be *p* ≤ 0.05

## 3. Results

### 3.1. The Effects of Experimental Factors on LAB Count

The effects of the experimental factors on LAB count are shown in Table 2 and Table 3. There was a highly significant effect of feeding group on LAB count (Table 2, *p* < 0.001). The LAB count was highest in the faeces of animals in Groups 3 and 2 (*p* < 0.05). The LAB counts of the faeces of goats in Groups 1, 2, and 4 were similar (*p* < 0.05). Excluding Group 4, the LAB content was significantly higher than in controls (*p* < 0.05). There was a highly significant effect of sampling time on LAB count (*p* < 0.0001, Table 2). The introduction of herbs into the diet of dairy goats increased the LAB count in the GI tract. There was a significant increase in the LAB count in stages T1 and T2 of lactation. The LAB count was highest in T2, the peak period of lactation, which indicates that there was significant effect of the stage of lactation on LAB count (Table 3). The LAB count determined in the faeces of Group 3 during the T2 measurement period was significantly higher than that in all other animal groups.

### 3.2. Identification of LAB Strains

Table 4 shows the results of identifying microorganisms from the DNA of the most frequently and morphologically repeated bacterial colonies.

The following species of microorganism were found in the genetic material isolated from the experimental and control samples: *Lactobacillus buchneri* strain JCM 1115, *Enterococcus faecium* strain ATCC 19434, and *Enterococcus mundtii* strain NBRC 100490. Furthermore, *Lactobacillus fermentum* strain NBRC 15885 was present in faecal samples collected from the goats in the experimental groups.

The genetic identification of faecal samples of the controls revealed, in addition to LAB, the presence of spores of the fungus *Aspergillus fumigatus* in the digestive tract of goats. This is a pathogenic exogenous fungal species that may cause various infections in animals. In ruminants, in addition to weakening of the immune system, this species can affect the throat, nasal mucous membranes, or lungs, and can cause acute enteritis.

No mould spores were detected in the samples from the goats fed with the herb supplements.

## 4. Discussion

LAB are gram-positive, nonsporulating bacteria with low guanine-cytosine (GC) pairs in the genome. This group was singled out for its ability to perform carbohydrate fermentation with production of lactic acid, rather than for its phylogenetic relationships [17]. Although most LAB are anaerobia, some species may tolerate low levels of oxygen. LAB have strong auxotrophy, and are thus found in environments that accommodate their high nutritional requirements—i.e., that are rich in amino acids, purines, and pyrimidines. LAB can be found in milk and its derivative products, and are also components of the physiological flora (microbiota) of mammals. This group of microorganisms includes species of the *Lactococcus*, *Streptococcus*, *Pediococcus*, *Leuconostoc,* and *Lactobacillus* genera. Probiotic species are particularly valuable LAB [18]. These bacteria modulate the gut flora and thus maintain its homeostasis. They provide protection against pathogenic bacteria by competing with them for colonised surface. LAB can secrete compounds that inhibit pathogen growth (lactic acid, short-chain fatty acids, hydrogen peroxide, and substances that act as bacteriocins) [17]. Moreover, they stimulate the immune system and reduce the risk of allergic reactions [17].

Probiotic strains play the most significant role in supporting the treatment of GI diseases—especially viral diarrhoea and antibiotic-associated diarrhoea—and autoimmune disorders [14,17]. Their positive effects on metabolic diseases (hyperlipidaemia, diabetes, and obesity) have also been observed [16]. Probiotics are also credited with alleviating symptoms of lactose intolerance, increasing intestinal absorption of nutrients, lowering cholesterol level, improving intestinal peristalsis, and decreasing the activity of enzymes associated with carcinogenesis [14].

The literature contains few results from research works concerning the microbial composition of faeces in ruminant animals, mostly focusing on dairy cattle. Experiments conducted on calves have shown the relationship between faecal microbiota and age [19], nutritional diet [20,21], antibiotic therapy [22,23,24], and calf health status [25].

The effect of limit-feeding diets with different forage-to-concentrate ratios on faecal bacterial community composition in Holstein heifers has been studied by Zhang et al. [26] and others. In the study of Kim et al. [27], concerning bacterial diversity in the faeces of cattle fed different diets, faecal samples were collected from cattle fed a finishing steer diet (“moderate grain diet”), a late growing diet (“high-grain diet”), and from heifers fed an early growing diet (“silage/forage”). The taxonomic composition of faecal microbiota in these three diet groups was compared based on the mean of the relative abundance (reads of taxon divided by total number of reads in the sample). The abundance of *Lactobacillus* was different (*p* < 0.001) in the three groups; the high-grain diet group had the greatest abundance (1.50% of total sequences).

There have been few reports on the effect of lactation stages in ruminants on faecal microbiota composition. The report of Huang et al. [28] shows a significant effect of lactation period on diversity at the phylum level in the faecal bacterial community. This means that lactation stages induce a variation in the faecal bacterial community [28].

There are very few results concerning the composition of the gut microbiota of goats. The study of Draksler et al. [29] is one of the few reports to describe the number density of LAB. The LAB content, identified in faecal samples of Creole goats kept in northwestern Argentina, reached its highest value in the first two weeks of a goat kid’s life and ranged from 5.58 to 7.15 log_10_ units/g of faeces [29]. In animals aged 30–60 days the CFU of LAB count decreased, reaching 5.24 and 5.43 log_10_ units/g of faeces, respectively. LAB content held stable from ninety days of age onwards. For animals in each age range, the log_10_ value of LAB was 4.61 (90 days), 4.93 (120 days), 4.82 (150 days), 4.52 (180 days), and 4.52 (270 days) [29].

Stella et al. [30] evaluated the effect of administering live *Saccharomyces cerevisiae* on milk production, milk composition, blood metabolites, and faecal flora in early lactating dairy goats. There was a significant effect on faecal flora. The differences between the control and experimental groups in terms of colony counts of *Lactobacilli* were particularly pronounced and statistically significant at sixty and ninety days of lactation, at 5.05 versus 6.21 and 4.89 versus 6.37 log_10_/g of faeces, respectively.

The following LAB species were identified in faecal samples: *Lactobacillus buchneri* strain JCM 1115 (experimental and control groups) and, only in the experimental group samples, *Lactobacillus fermentum* strain NBRC 15885. Both L. *buchneri* and L. *fermentum* are typical probiotic bacteria with proven antioxidant activities. According to the experiment conducted by Shokryazdan et al. [31], these strains had good antimicrobial activity against selected pathogenic strains of humans and exhibited stronger antimicrobial activity than the reference strain, L. *casei* Shirota.

The genetic identification performed as part of our experiment also revealed the presence of microbes such as *Enterococcus* *faecium* strain ATCC 19434 and *Enterococcus faecium* strain NBRC 100490. *Enterococcus* is a genus that is commonly found in the gut microbiota of ruminant animals, especially in the first stages of life that are not related to rumination. Jiao et al. [32], who studied the gut microbiota of goat kids during their first week of life, estimated the proportion of *Enterococcus* in the total species composition of the gut microbiota at 30.94% of the sequences under study.

It should be noted that spores of an exogenous fungus of the pathogenic species *Aspergillus fumigatus* were identified in goat faeces collected from controls. In ruminant animals this species may lead to various infections and even acute enteritis under extreme conditions [32].

## 5. Conclusions

There is a significant effect of the herbal feed additive on LAB count (*p* < 0.001). The highest number density of LAB was found in the group of goats receiving a feed additive that contained nine herbs at 20 g/animal per day (*p* < 0.05).

There was a statistically strong effect of lactation stage on intestinal LAB count (*p* < 0.001) The greatest number density of LAB was found in animals of all feeding groups at the peak of lactation (T2). Moreover, there is a highly significant interaction of feeding group × time of faecal sample collection (*p* < 0.0001).

The valuable probiotic species *Lactobacillus fermentum* strain NBRC 15885, is present in faecal samples of goats receiving a herbal additive compared to controls. The results of the genetic identification of faecal samples collected from the animals receiving the herbal supplement did not reveal the presence of mould spores, which are potentially harmful to the health of small ruminants; however, these spores were identified in controls.

## 6. Patents

A patent for “Herbal feed additives and their application” was submitted to the Polish Patent Office for intellectual protection of this technology (Polish Patent Application No. P.433779 and P.434426).

## Figures and Tables

**Table 1:** The composition of the experimental concentrates (% DM).

	Dietary Treatment		
Item	Groups 1 and 3	Groups 2 and 4	Group 5 (Control)
Ingredient (% DM)			
Wheat bran	17	13	17
Triticale	18.6	18	18.6
Rapeseed meal	17	16.5	17
Sunflower meal	10	9.5	10
Corn DDGS ^a^	5	5	5
Rye	7	6	7
Wheat	5	5	5
Barley	4	4	4
Dried grasses	0	0	6.6
Herbs	6.6	13.2	0
Sugarcane molasses	2	2	2
Dried sugar beet pulp	4.2	4.2	4.2
Minerals and vitamins ^b^	2.5	2.5	2.5
Fodder chalk	0.1	0.1	0.1
Salt	1	1	1
Composition (g kg^−1^ DM)			
Organic matter	927	926	928
Crude protein	229	223	224
Crude fat	36	33	34
Crude fibre	86	92	87

Groups 1 and 2: a mix of seven herbs; groups 3 and 4: a mix of nine herbs; group 5: control group (no herbal supplements); ^a^ corn DDGS, distiller’s dried grain with solubles from the production of biodiesel and ethanol; and ^b^ 1 kg of minerals and vitamins contains 300,000 units of vitamin A, 30,000 units of vitamin D_3_, 1.5 g of vitamin E, 0.5 g of Fe, 2.5 g of Zn, 65.0 g of Mg, 0.015 g of Co, 3.0 g of Mn, 0.01 g of I, 0.003 g of Se, 60 g of Na, 240 g of Ca, and 120 g of P.

**Table 2:** Effects of experimental factors on LAB count.

LAB	Group					Time				SE	Group	Time	Group × Time
	1	2	3	4	5	T0	T1	T2	T3		*p*-Value		
Transformed CFU	6.82 ^a^ 3.15 × 10^8^	6.93 ^ab^ 2.37 × 10^9^	7.03 ^b^ 1.92 × 10^9^	6.75 ^ac^ 1.95 × 10^9^	6.60 ^c^ 6.92 × 10^5^	6.35 ^a^ 9.40 × 10^4^	6.80 ^b^ 7.03 × 10^8^	7.24 ^c^ 4.54 × 10^9^	6.91 ^b^ 4.97 × 10^6^	0.03 -	0.0001 -	0.0001 -	0.0033 -

Means marked with different letters are statistically different at *p* ≤ 0.05. Transformed: value after Box–Cox transformation (first row); CFU: number of colony-forming units (second row); and SE: standard error.

**Table 3:** Effects of measurement time on LAB count in groups.

LAB	Group	Time				SE
		T0	T1	T2	T3	
Transformed CFU	1	6.35 ^a^ 9.33 × 10^4^	6.82 ^b^ 1.51 × 10^6^	7.34 ^c*ef*^ 1.25 × 10^9^	6.77 ^b^ 2.58 × 10^6^	0.07 -
Transformed CFU	2	6.37 ^a^ 9.41 × 10^4^	7.01 ^b^ 2.36 × 10^9^	7.35 ^b*ef*^ 7.13 × 10^9^	6.98 ^b^ 4.55 × 10^6^	0.08 -
Transformed CFU	3	6.36 ^a^ 9.51 × 10^4^	6.98 ^b^ 4.28 × 10^7^	7.72 ^c*e*^ 7.63 × 10^9^	7.07 ^b^ 1.19 × 10^7^	0.09 -
Transformed CFU	4	6.33 ^a^ 9.33 × 10^4^	6.65 ^ab^ 1.11 × 10^9^	7.11 ^b*fg*^ 6.69 × 10^9^	6.90 ^b^ 4.03 × 10^6^	0.08 -
Transformed CFU	CTRL	6.36 ^a^ 9.41 × 10^4^	6.55 ^ab^ 5.39 × 10^5^	6.66 ^ab*g*^ 4.25 × 10^5^	6.81 ^b^ 1.71 × 10^6^	0.05 -

Means marked with different letters are statistically different at *p* ≤ 0.05. Reading across rows, the letters mark the significance of differences within experimental groups. Reading down columns, the letters mark the significance of differences among experimental groups by time of measurement (*italics*). Transformed: value after Box–Cox transformation (first row); CFU: number of colony-forming units (second row); and SE: standard error.

**Table 4:** Alignment of consensus sequence for the 16S query fragments with the subject sequence in samples collected from animals of the experimental and control groups.

Species of Bacteria	Similarity	Sequence Coverage
Experimental and control groups		
*Lactobacillus buchneri* strain JCM 1115	99.8%	100%
*Enterococcus faecium* strain ATCC 19434	100%	100%
*Enterococcus mundtii* strain NBRC 100490	100%	100%
Experimental group		
*Lactobacillus fermentum* strain NBRC 15885	99.9%	100%
Control group		
*Aspergillus fumigatus* isolate C1946	100%	100%

## Data Availability

The data presented in this study are available on request from the corresponding author.
